# Productive, Physiological, and Environmental Implications of Reducing Crude Protein Content in Swine Diets: A Review

**DOI:** 10.3390/ani14213081

**Published:** 2024-10-25

**Authors:** André Martinho de Almeida, Maria Angeles Latorre, Javier Alvarez-Rodriguez

**Affiliations:** 1 LEAF—Linking Landscape, Environment, Agriculture and Food Research Center, Associated Laboratory TERRA, Instituto Superior de Agronomia, Universidade de Lisboa, Tapada da Ajuda, 1349-017 Lisboa, Portugal; 2 Departamento de Producción Animal y Ciencia de los Alimentos, Facultad de Veterinaria, Instituto Agroalimentario de Aragón-Universidad de Zaragoza, Calle Miguel Servet 177, 50013 Zaragoza, Spain; malatorr@unizar.es; 3 Departamento de Ciencia Animal, Universidad de Lleida, Av. Rovira Roure 191, 25198 Lleida, Spain

**Keywords:** nitrogen excretion, ideal protein balance, pig production, ammonia, amino acid supplementation

## Abstract

Pig production is one of the most important providers of high-quality proteins and amino acids (AAs) to human nutrition. In this sector, feeding has an important economic and environmental impact. A strategy to reduce production costs and negative sustainability effects is reducing dietary crude protein (CP) contents with or without AA supplementation. This review addresses the different aspects related to this strategy, particularly the effects on growth performance and pork traits in piglets and growing and finishing pigs, as well as the physiological molecular mechanisms’ underlying effects. Insight is also provided into the effects of dietary CP reduction on the productive performances of alternative pig production systems and breeding boars and sows. Finally, an overview is conducted on the effects of dietary CP reduction on ammonia, odor, and greenhouse gas emissions arising from pig production systems. Overall, CP reduction may lead to production losses, albeit they can be, to some extent, hindered by adequate AA supplementation. Losses are particularly relevant during the post-weaning phase, whereas in finishing pigs, it may bring additional benefits, such as high intramuscular fat contents in some markets or improved gut barrier function with benefits to the animals’ health and welfare, as well as decreased ammonia emissions to the environment.

## Simple Summary

Nutrition represents 70–80% of pig production costs, particularly related to protein and amino acids, which are also crucial for human nutrition. Proteinaceous feedstuffs are generally imported from North and South America to Asia and Europe at high costs and with negative environmental effects. Reducing dietary crude protein (CP) contents with or without AA supplementation is an interesting strategy to reduce production expenses and adverse environmental effects. Herein, we conduct a literature review on the productive, physiological, and environmental effects of the strategies across different production systems and types of animal in the pork production chain. CP reduction leads to production losses that can nonetheless be curbed by adequate AA supplementation, particularly in growing and finishing animals.

## 1. Introduction

The world’s human population is expected to increase to 10 billion by 2050 [1], particularly in developing countries. To feed such a large population, agricultural outputs will have to increase substantially, placing a significant strain on agricultural land and resource usage, raising, in turn, sustainability issues [2]. A balanced human diet requires foodstuffs of animal origin, namely dairy, eggs, or meat. The latter are particularly important for the supply of dietary crude protein (CP), essential amino acids (AA) and lipids, as well as minerals and vitamins [3]. In a livestock production context, pork and poultry meat hold affordable animal proteins and AAs for the human diet. These two sectors will thus play a pivotal role in adequately feeding such a large human population [4].

Modern pork production is a very specialized and optimized industry that uses state-of-the-art production methods in every aspect of the chain: genetic improvement, reproductive and health management, facilities and environmental control, slaughter, processing and marketing, effluent management, and nutrition and feeding [5]. The latter are responsible for roughly 70–80% of the production cost and, within a context of decreasing profit margins, have to constantly be optimized according to the volatility of supply and demand for the different foodstuffs used in pig production [6]. High-nutritional-value proteins (i.e., with an adequate composition of essential AAs) represent a significant proportion of the feed production cost, and thus affect the whole chain and ultimately condition the final price to the consumer. Reducing the CP contents of pig feed would thus be an interesting strategy towards the economic sustainability of pork production, with the additional benefits of putatively lowering the emission of greenhouse gasses (GHG) in pig slurry [7] as well as ammonia emissions and pollutant characteristics [8]. Furthermore, it will contribute to a reduction in acidification, eutrophication, and odor impacts of pig manure [9].

An interesting possible optimization of the use of nutritional resources, particularly protein, in pig nutrition would simply be feeding the pigs with below-standard CP levels. This can be achieved by reducing soybean meal incorporation and increasing the use of synthetic AA. These include lysine (Lys), methionine (Met), threonine (Thr), tryptophan (Trp), valine (Val), isoleucine (Ile), histidine (His), and arginine (Arg). This is particularly important in a context where prices of such AAs are becoming more competitive, contrary to those of standard foodstuffs [10].

Lowering the feed CP content can have significant consequences on pig performance [11], even when diets are balanced for all essential AAs, although results vary considerably in the literature [12]. In fact, the NRC [13] establishes a minimum dietary nitrogen (N) concentration in addition to essential AAs, as it has been shown that pigs fed low-CP diets may need more N for endogenous synthesis of non-essential AAs to support protein synthesis [14]. Taking into account the fact that energy intake limits the protein deposition during the early phase of growth [15], the digestible AAs to metabolizable energy ratio may have to be increased when dietary CP is reduced.

The AAs are traditionally classified as essential (in alphabetic order: His, Iso, Leu, Lys, Met, Phe, Thr, Trp, and Val) or nonessential, depending on whether they can be synthesized by the pig and can support the N balance of the organism. However, there is growing evidence in the literature that has led to the development of a novel concept of functional AA in nutrition, being that they, albeit defined as nonessential AAs, regulate key metabolic pathways to benefit survival, growth, development, reproduction, lactation, and health (e.g., Arg, Cys, Gln, Glu, Gly, and proline Pro). Functional AAs can be nutritionally essential, nonessential, or conditionally essential [16]. Pig diets are traditionally formulated to contain ideal ratios of essential AAs (with Lys as the reference AA) based on their concentrations in the pig carcass (almost exclusively tissue proteins) [17] or milk [18]. However, this feed formulation approach may neglect certain non-essential AAs which are not yet evaluated in the dietary ingredient matrix.

Reducing dietary CP is thus a debatable strategy, particularly from an economic perspective where growth and feed conversion ratio losses arising from CP content reduction would have to be matched by the savings in feeding costs. This subject has been the object of a meta-analysis study regarding the productive implications and consequences of reduced CP content in pig nutrition [12], mainly concluding that there is a minimum CP level after which the growth performance of pigs is compromised, even when diets are balanced for essential AAs.

The objective of this work is to conduct an extensive literature review on the productive, reproductive, physiological, and environmental implications of reducing CP contents in swine diets. This review provides a contemporary approach to the subject and includes updated findings by groups working in the field. It aims to provide an updated and focused insight about this issue in all aspects of pig nutrition, including different production stages in industrial pig production (post-weaning, growing, and finishing pigs), as well as boars, sows, and pigs raised under alternative production systems like those in Southern Europe. The ultimate goal of this work is to contribute with updated knowledge on the effects of CP reduction and AA supplementation under the ideal protein concept as a guideline for animal and nutrition scientists as well as veterinarians and technicians working in the field. The review is divided in six major sections: (i) this background to contextualize the topic; (ii) the consequences of CP reduction in the productive parameters of piglets and growing and finishing pigs of modern standard pork production lines; (iii) the consequences of CP reduction in the productive parameters of pigs in alternative production systems, particularly extensive, organic, and premium pork; (iv) the implications CP reduction has on productive and reproductive parameters in boars and sows; (v) the physiological consequences of CP reduction in all types of pigs, with a special emphasis on changes occurring at the molecular level; and (vi) the implications CP reduction has on the environmental load and overall sustainability of the system. The manuscript ends with major conclusions and future prospects on the subject, and, finally, addresses the consequences of CP reduction in feeds across the different components of the pig production sector. In this last section, practical recommendations are issued that focus on this strategy and its implications for swine nutrition.

## 2. Effects of CP Reduction on the Productive Parameters of Piglets and Growing and Finishing Pigs of Lean Genetic Types

**Figure 1:**
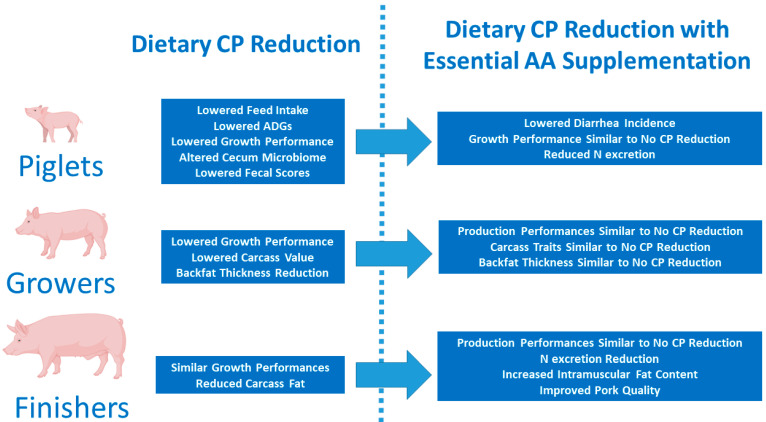
Major effects at the productive level of a reduction from 2 to 5% dietary CP, with or without crystalline amino acid supplementation in piglets and growing and finishing pigs of lean genetic types.

Pigs raised for pork meat production undergo three major stages: (a) piglet, (b) grower, and (c) finisher. Animals in these three stages have very different nutrient requirements, particularly regarding CPs and AAs, in order to fully achieve their genetic potential regarding growth and feed conversion. The first stage is the most critical, as piglets have an underdeveloped immune system and are subjected to numerous stress factors, namely the transition from milk to solid feeds, as well as environmental changes inherent to moving away from sow, the adaptation to different facilities, littermates, and increased microbial challenges. Furthermore, piglets and growers have high CP and AA requirements, given the fact that these animals are at the initial phases of the growth curve and, as such, have very high levels of protein synthesis and development/growth of muscle tissues. Nonetheless, they have a limited voluntary feed intake to sustain such high levels of protein synthesis and development/growth of muscle tissues at the initial phases of the growth curve. On the contrary, finishing pigs are at a different phase where their live weight starts to stabilize, skeletal muscle growth decreases, adipose tissue growth starts to increase substantially, and, furthermore, their voluntary feed intake levels increase. Lowering the CP contents of feeds would thus be more applicable for finishing pigs, where their needs are not as critical. Finishing pigs are much heavier than growing ones. Therefore, a reduction in the CP content of feeds could lead to a reduction in the feeding costs of these animals, albeit this very dependent on AA concentrations and prices [11,14]. Another important factor that influences dietary CP and AA requirements is the sex of the animals, especially after 50 kg of body weight (BW), with males having higher needs than gilts or barrows [13]. Interestingly, and as far as CP reduction is concerned, the piglet phase is perhaps the most studied. Figure 1 summarizes the main effects of dietary CP reduction with or without crystalline AA supplementation, as described subsequently.

The effects of lowered CP contents have been addressed since the mid-1980s. McCracken and McAllister [19] studied the influence of energy metabolism and body composition in Large White × Landrace piglets fed between 17, 21, 46, and 225 g CP/kg in their diet. The authors highlighted a decrease in daily heat production, as well as a decrease in carcass CP gain and CP balance and an increase in fat gain, alongside the decrease in CP intake [19]. This study finally indicates that heat production declines when the growth rate of young pigs is reduced due to energy and protein restriction, which does not support the theory that low-protein diets give rise to increased diet-induced thermogenesis [19]. More recently, Luo et al. [20], working with Duroc × (Landrace × Large White) piglets, also demonstrated a negative effect of a 6% CP reduction (20 to 14%) on growth performance (lower feed intake and ADG—Average Daily Gain—with a higher FCR—Feed Conversion Ratio) as well as altered microbial communities in cecum contents. Similarly, a reduction from 24 to 18 and 12% CP in crossbred piglets led to poorer growth performances in the latter nutritional groups [21]. A similar conclusion regarding productive performance was obtained with piglets fed low (22, 16, and 15%) CP-content diets, although reduced-CP animals had lowered fecal scores, improved feces consistency, and a downregulation of inflammation-associated genes [22].

AA balancing, particularly Lys, is an interesting way of minimizing the negative effects of CP reduction on piglet productive performance. Heo et al. [23] compared high- (240 g/kg) to low-CP (180 g/kg) diets with balanced AAs in post-weaning Large White × Landrace piglets, finding that low-CP diets led to decreased plasma urea N and fecal ammonia-N contents, as well as a reduced incidence of post-weaning diarrhea. Interestingly, low-CP diets did not reduce growth performance up to 106 days after weaning compared to pigs fed the high-CP diet, a possible consequence of the fact that the diets were balanced for all essential AAs [23]. Gloaguen et al. [24] reached similar conclusions regarding the growth performances of barrow and female Pietrain × (Large White × Landrace) piglets subjected to different dietary CP levels, reporting that a CP reduction from 19.7 to 16.8% led to a decreased N excretion by 29% but had no influence on N retention, unlike the lower levels (between 14.0 and 12.7%). These authors also discovered that a 4% CP decrease from 17.6 to 13.5% also had no effect on the performance of these animals, demonstrating that cereals and specific AA supplementation could ultimately replace soybean meal. Zhou et al. [25] reached an analogous conclusion, demonstrating that the negative effects of a CP content reduction from 20 to 18.5% in Duroc × (Landrace × Yorkshire) piglets could be countered by increased levels of Lys. Millet et al. [26] conducted a study with different CP contents (140, 150, 160, 170, 180, and 190 g/kg) with two levels of standardized ileal digestible (SID) Lys and established that the productive effects of decreased CP contents in piglets is ultimately dependent on SID Lys levels. Liu et al. [27] carried out a similar approach, comparing Duroc × (Landrace × Yorkshire) piglets fed CP levels between 20 and 17% with the same levels of Lys; animals in the former group had improved growth traits, albeit with similar FCRs. In a second experiment, with the same CP levels but Lys supplementation at lower CP contents, the authors discovered that Lys supplementation could also counterbalance the negative effects of low-CP content. On the contrary, Lynegaard et al. [28] found that recently weaned piglets [Duroc × (Danish Landrace × Yorkshire)] fed diets with very low CP contents (151 g/kg), albeit corrected for essential AAs, had poorer growth performances than the control piglets, although there were clear benefits, such as reducing diarrhea incidence in the former group. The authors, furthermore, proposed low-CP diets as a measure to circumvent the negative production effects of ZnO withdrawal. Marchetti et al. [29] obtained a similar conclusion in Duroc × Large White piglets fed with 2% CP reductions (from 17.5 to 15.5% CP) with balanced AA levels, reporting that the animals had similar growth performances and lower fecal scores. Other studies showed worse ADG and/or FCR when reducing CP levels from 21 to 18% [30] and from 19 to 16% [31], balancing AA in both cases. On the contrary, a moderate reduction in dietary CP contents (from 18 to 15%) with AA balancing in cross-bred Sushan piglets led to similar growth performances, with improved results regarding intestinal microbiota [32]. It needs to be stated, however, that this research was conducted with a pig breed that had a 25% component from a Chinese pig breed, thus having fattier carcasses and, therefore, lower CP and AA needs in comparison with European lean breeds [33].

In addition, recent results suggest that N may be limiting in diets with a high essential AA to total N ratio. Therefore, to maximize N retention in growing barrow pigs from modern crossbreds, it would be warranted to reduce the essential AA/total N ratio while increasing the dietary Lys supply [34].

The supplementation of other specific essential AAs has also been studied in piglets. Ren et al. [35] conducted an approach comparing Duroc × (Landrace × Yorkshire) piglets fed high (21%) and low (17%) CP contents, as well as the effect of BCAA (branched chain AAs) supplementation (namely Iso, Val, and Leu), with all diets showing similar levels of essential AA contents. Reduced CP content reduced feed intake, increased FCR as well as villous atrophy, and increased intra-epithelial lymphocytes counts, finally demonstrating that BCAA could compensate for the negative effects of lower CP contents. A similar conclusion was found in an experiment on the beneficial effects of BCAA supplementation in Large-White × Landrace barrows fed low-CP diets [36]. Similarly, Upadhaya et al. [37], studying the effect of low-CP diets supplemented with glutamic acid in Duroc × (Yorkshire × Landrace) piglets, determined that glutamic acid could counterbalance the negative effects of a low-CP diet on animal growth performance. In a study of a very different nature evaluating potential interactions with parasite load, dietary CP levels between 28, 20, and 16% had little effects on protection from trypanosome infection in piglets [38].

From what was previously stated, one could infer that the effects of low CP on the growth performance of piglets leads to divergent results. Ultimately, productive performances are dependent on the CP reduction’s magnitude, crossbreds, sex, and AA supplementation, particularly of Lys and other essential AAs, although the benefits of supplementing specific AAs may also be beneficial for piglet performance.

In growing–finishing pigs, the results tend to be more consistent than in piglets. Indeed, with no AA balancing, growing and finishing pigs fed with low CP contents tended to have lower growth performance and carcass values [39], although these effects could be reverted by directed AA supplementation, particularly of Lys, Trp, and Thr. On the contrary, when AA supplementation was used, Kerr et al. [40] observed that overall production results were not impaired when providing 19, 16, or 14% CP to pigs. Similar conclusions [41] were drawn regarding backfat thickness measurements of gilts when decreasing CP levels were implemented in the growing phase (between 16, 14, and 12%) and in the finishing phase (between 14, 12, and 10%). Other authors did not detect a penalization on pig performance irrespective of space allocations (with different CP levels for growers—14 vs. 17%—than for finishers—12 vs. 15%-) [42] and sex [43]. More recently, Hong et al. [44], working with Duroc × (Yorkshire × Landrace) pigs, also observed the absence of an effect from low CP contents on the growing and finishing stages on growth performances, although there was a trend for backfat thickness reduction, which showed an influence on pork quality (decreased water holding capacity and increased cooking losses as a consequence of CP reduction). Corroborating these results, Monteiro et al. [45], whose research used barrows and gilts [Pietrain derived × (Landrace × Large White)] and a 3–4% CP reduction with AA balancing at different stages, found no differences regarding growth performances and carcass traits, other than the expected sex-related differences. The different responses across studies may be related to the in-feed AA supplementation level needed to meet requirements. Throughout the growing and finishing periods, the limitation order of essential AAs is dynamic. While Lys is the first limiting AA at all times, Met is the second limiting AA at 25–50 kg, but is the fourth limiting AA onwards, whereas Thr becomes the second and Trp the third limiting AA from 50 kg onwards. In low-CP diets, throughout the growing and finishing periods (from 13.5 to 9.3%), Val is more required than Ile in the early growing phase (25–50 kg), while Ile becomes more required in the late growing and finishing phases (75–125 kg), but becomes the fourth limiting AA in this last part of the finishing period [46]. More recently, Mun et al. [47] conducted a study using Duroc × (Landrace × Yorkshire) crosses and low-CP diets (15.7%) and Lys supplementation levels ranging from 1.10 to 1.15% at the start of the growing period (17 kg of body-weight), with similar results at the end of a 52-day trial to those obtained using controls without Lys supplementation and higher dietary CP (17.2%).

Low CP dietary contents ultimately led to decreases in N excretion and balance, as well as lower feed production costs and GHG emissions [45]. Similar results were obtained by Vonderohe et al. [48] with Duroc × (York × Landrace) growing–finishing pigs under adequate and reduced CP content feeds with AA balancing. According to the latter authors, reductions in dietary CP with crystalline AA supplementation resulted in an overall reduction in total N and mineral excretion, and animals fed with the lower CP levels had reduced performance and carcass characteristics, unlike animals in the intermediate CP levels. Interestingly, and in addition to a general first-limiting AA balancing, other compounds have been found to mitigate the detrimental effects of extremely low-CP feeds on growing pig performance, for instance, casein hydrolysate [49] or specific AA supplementation such as glutamic acid [50] or Val and Ile [51]. Finally, an interesting beneficial decrease in the incidence of damaging behaviors may also be observed in low-CP–AA supplemented pigs [52].

Specifically, in finishing pigs, the subject has also been addressed, albeit at a lower level than in piglets and growing animals. Knowles et al. [53] determined similar growth performances and reduced carcass fat when comparing diets with crystalline AA and low CP to control diets in finisher (Yorkshire × Landrace × Hampshire) pigs. A similar approach was conducted on PIC barrows when reducing CP but with AA balancing, and it was observed that N excretion decreased without influencing growth performance and that adding fiber to the diet was an interesting way to reduce slurry ammonium N concentrations [54]. This is consistent with results that show higher N excretion with 200 g CP/kg in comparison to 150 g CP/kg diets if they were AA balanced and inulin supplemented [55]. It has been suggested that feeding fermentable fiber to pigs can shift the balance of N excretion from urine to feces [56,57] by binding N into microbial protein in the large intestine [58]. However, it has been found that the effects of dietary CP and fiber were independent of each other [59]. Indeed, reducing dietary CP (from 198 to 172 g/kg) and adding crystalline AA to the feed did not affect the growth performance of Large White × Landrace pigs from 6 to 11 weeks of age (from 14 to 38 kg BW). Thereafter, however, to avoid impairing production parameters and excess carcass fatness in pigs from 12 to 21 weeks of age (from 38 to 110 kg BW), it was more feasible to increase dietary fiber with constant energy density. An increase in dietary fiber in low-CP feed did not modify the amount of CP and fiber digested, which suggests that this dietary manipulation would only be useful for shifting the balance of N excretion from urine to feces at high dietary CP levels. In addition to growth performances, low-CP diets have also been found to increase the intramuscular fat (IMF) content, backfat thickness, and overall pork eating quality, albeit decreasing loin weight in (Duroc × Pietrain) × (Large White × Landrace) finishing entire animals [60]. More recently, Apple et al. [61] studied the effect of low-CP AA balanced diets on growth performance, carcass composition, and pork quality of (PIC 380 × GPK-35) finishing pigs. Dietary CP reductions led to decreased ADG and average daily feed intake, had no effects on carcass yield, backfat depth, or ham composition, and increased marbling and IMF content. Finishing pigs seem to be the type of pigs where CP reductions balanced by AA supplementation seem to be the most effective. In accordance, Song et al. [62] conducted a study with Duroc × (Landrace × Yorkshire) finishing pigs. These authors used different levels of CP (early finishing: 150, 142, 134, and 126 g/kg; late finishing: 140, 130, 120, and 110 g/kg). The authors established that, with an adequate AA balance (using all of the available commercial AAs: Lys, Met, Thr, Trp, Val, Ile, and His), CP contents could be reduced to as low as 126 g/kg and 120 g/kg, respectively, in the early (up to 80 kg BW) and late finishing (up to 120 kg BW) periods under commercial conditions and without compromising productive performances. Consistently, Liu et al. [63] studied the effect of a 4% CP content decrease in Duroc × (Landrace × Yorkshire) finishing pigs and observed that the CP reduction led to increased IMF content, different muscle fatty acid and AA composition, and cecum microbiota profiles with similar growth performances. Overall, and in comparison to piglets and growing pigs, finishers are the type of animal for which dietary CP reduction, with adequate AA balance, is more adequate, leading to performances similar to those of animals fed standard dietary CP levels, with several benefits, furthermore, regarding lowered N excretion and improved pork quality.

## 3. Effects of CP Reduction on Productive Parameters in Alternative Production Systems

In Southern European countries, i.e., Spain and Portugal, alternative (or extensive) production systems are very important for the production of high-value pork products such as sausages or cured hams. Those systems use autochthonous breeds, like the Iberian/*Alentejano*, whose pigs are typically raised until much older ages and heavier BWs when compared to modern standard pork production hybrids slaughtered at 100–120 kg. Analogous systems also exist in other regions of the Mediterranean, such as Italy, particularly directed toward the production of premium hams. Other alternative systems also include organic pork products that are produced in free-range outdoor systems, particularly in Northern Europe. In all of those systems, pigs are grown until older ages than in conventional production systems. Furthermore, slower growth/fattier breeds or cross-breeds are used, and animals are fed not only on commercial feeds, but also on pasture foraging and particular seasonal resources, such as Mediterranean Oak acorns. In those systems, ADGs are slower and do not support adipose tissue accretion, which is more energy consuming than muscle synthesis. Accordingly, CP and AA supplementation is not as critical as in conventional pig production systems.

**Figure 2:**
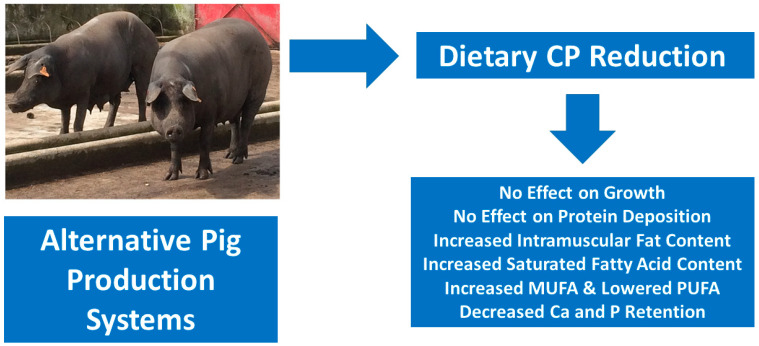
Major effects at the productive level of a dietary CP reduction in heavy Iberian pigs produced under alternative systems in the Iberian Peninsula.

In Iberian pigs, it has been found that calcium and phosphorus (P) retention was lowered as a consequence of lower levels of dietary CP (192 vs. 101 g/kg), although the growth rate and protein deposition were not affected [64]. These results are in accordance with others that studied the effect of two CP levels in Duroc × Iberian cross-breeds, particularly during the last stages of production [65]. In both Alentejano and Pietrain × (Large White × Landrace) cross-breeds, Madeira et al. [66] studied the effect of reducing the CP diet from 17.5 to 13.1%, determining that the lower CP levels increased the IMF content (+15.7%) and the proportion of monounsaturated fatty acids (MUFA) (+5.2%) and saturated fatty acids (SFA) (+3.2%), but decreased polyunsaturated fatty acids (PUFA) (−14.8%). Tejeda et al. [67] confirmed this finding more recently, reporting that higher IMF contents (+26.6%), as well as low muscle and fiber areas, had higher SFA (+8.0%) and lower PUFA proportions (−16.3%) in castrated Iberian pigs slaughtered around 175 kg after feeding on a diet with 6.6% CP when compared to pigs fed on a 12.8% CP diet. On Duroc-crosses used in the production of Teruel dry-cured ham, despite the losses in growth production, CP restriction has been found to promote wider backfat depth and higher IMF contents with higher MUFA and lower PUFA contents, and thus with more interesting carcass and meat traits, in both barrows and gilts and in the production of dry-cured pork products [68]. The major effects of a dietary CP reduction in heavy Iberian pigs produced under alternative systems are summarized in Figure 2.

In a different production system, using heavy pigs of Italian breeds used for dry-cured ham production, Gallo et al. [69] tested decreased-CP diets (between 140, 128, 120, and 113 g/kg) supplemented with different AAs (from 6.5 to 5.3 g of Lys/kg) and found no effect on productive performance. Dietary CP reduction, furthermore, led to decreased production costs and reduced N excretion, ultimately allowing for an increase in the number of pigs per land area. The same research team also established that low-CP feeds had no influence on carcass weights, backfat thickness, and the weight of loins and dressed hams when compared to control animals [70]. These authors also established that a 20% reduction in both CP and Lys led to a 15% increase in the thickness of subcutaneous fat, a 5% decrease in linoleic acid and PUFA proportions in subcutaneous fat, and overall positive effects on the technological properties of hams used in PDO dry-cured ham production [71]. In Italian heavy pig breeds, dietary low CP contents, furthermore, had minor effects on the physical, chemical, and sensory characteristics of dry-cured Parma hams [72,73].

In organic pork, this topic has also been studied, although, in this particular context, production systems tend to be more varied than Southern European extensive systems, and in-feed AA supplementation is banned by EU production rules (Regulation (EU) 2018/848) [74]. In organic production systems, using [Pietrain × (Landrace × Large White)] crossbreds, a low-CP diet (206 vs. 183, 191 vs. 163, and 176 vs. 143 g/kg from 18 to 42, 42 to 71, and 71 to 113 kg BW, respectively) leads to poorer growth performances and FCRs during the first phase of growth (<45 kg BW). It also lead to lower meat percentages in the carcasses, but has a minimal effect on the meat’s traits [75]. The authors thus demonstrated the suitability of a low-CP diet on organic pork production in the Belgian context. It must be stated, however, that in the aforementioned study, even though the reduction in dietary CP led to increased backfat thickness in the carcass, the IMF of meat did not differ across diets. Indeed, this dietary manipulation does not turn into steady responses in the different carcass adipose tissue deposits.

Overall, it can be stated that low-CP feeding strategies are particularly interesting for alternative pig production systems. These include both organic and specialized premium pork production systems that use animals with lower growth rates and where growth performance is not as critical as in standard pork production systems. A low-CP feeding strategy, when adequately supplemented with AAs (except in organic production), leads, furthermore, to similar production performances, with little effects on both carcass and meat traits. This is particularly interesting for these systems, as they produce a high-value and high-quality product to an upscale market.

## 4. Implications of CP Reduction on Productive and Reproductive Parameters in Boars, Gilts, and Sows as Breeding Stock

**Figure 3:**
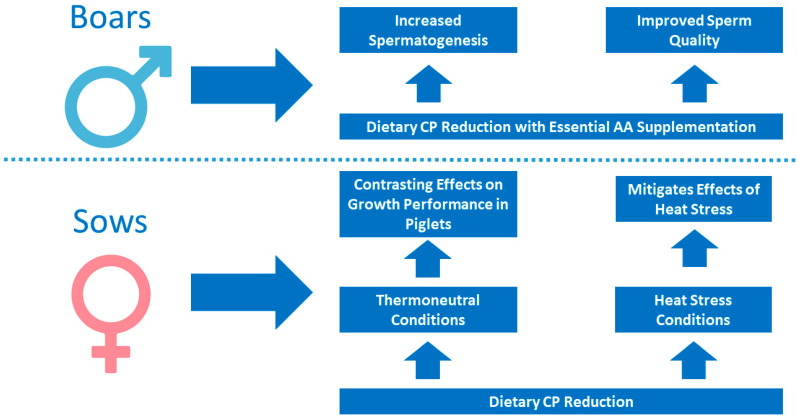
Major effects of a dietary CP reduction with crystalline AA supplementation in boars, and major effects of a dietary CP reduction under thermoneutral and heat stress conditions in sows. It is noteworthy to point out that for these classes of animals, the results found in the literature are very limited and often show the opposite effects.

The effect of dietary CP reduction has also been studied in breeding boars, gilts, and sows. In those animals, practical CP reduction is more difficult to implement, given the putative negative consequences on reproductive traits such as fertility, embryo survival, or milk production. Nevertheless, it could still be of interest in order to reduce feeding costs and N excretion from farms that would ultimately allow for a higher number of animals per farm and space. Figure 3 summarizes the main effects of a dietary CP reduction and crystalline AA supplementation strategy in boars and sows, as subsequently detailed in this section.

Studies in boars are very limited. Indeed, and to the best of our knowledge, only one study [76] addressed this topic. The authors studied the effects of Thr and Trp supplementation in low-CP diets on sperm and testis characteristics in (Landrace × Yorkshire) boars. The authors found that an optimum ratio of Lys:Thr:Trp:Arg of 100:76:38:120 in a 13% CP diet can lead to similar or even better reproductive performances than the control diet, which had 17% CP. Additionally, a diet with the same CP levels (13%) with different AA patterns has a strong influence on sperm quality, which may upregulate gene expression in testis cells to improve spermatogenesis.

In sows, different studies have addressed the effects of low CP levels. Earlier works address the beneficial effects of low CP under heat stress and piglet survival and performance. Johnston et al. [77] established that the growth rate of piglets was depressed when sows were fed a low-CP diet (16.5 vs. 13.7%) under warm (17.7–20.4 °C) conditions, but was conversely improved when sows were fed a low-CP diet under hot conditions (27.1–29.2 °C), but no effects were found for litter sizes. Other researchers also evaluated the impact of adequate and low CP contents (17.6 vs. 14.2%) at 20 vs. 29 °C in sows, finding that low-CP diets led to similar performances and reduced N excretion (−22.5%) in the lactating phase at thermoneutral conditions [78]. At high temperatures, it attenuated the negative effects on BW losses, although it did not affect reproductive performances. Consistently, Zhang et al. [79] observed a beneficial influence on lactating sows fed a low-CP (13.8%) AA-supplemented (Lys, Val, Thr, Phe, Met, Ile, His, Trp, and Leu) diet, based on higher apparent energy efficiency for milk production, when compared to a diet with 18.8% CP. These authors also highlighted the special effect of the additional Leu on the mitigation of heat production associated with lactation.

Under normal temperature conditions, this subject has also been addressed in different studies. The results tend to be quite varied, but generally indicate that, in pregnant and lactating sows, the negative effects of CP reduction can be balanced by adequate AA supplementation. If AA supplementation is conducted (Lys, Thr, and Trp), the reduction in dietary CP contents—for instance, from 14 to 12% in lactating sows—has been shown to have no effect on BW, backfat thickness, and apparent total tract digestibility of nutrients, but ultimately leads to poorer ADGs in piglets [80]. In this respect, Pan et al. [81] studied the effects of low-CP (18 vs. 15%) diets in sows during lactation on subcutaneous fat deposition in weaning piglets, demonstrating that maternal CP restriction without AA supplementation leads to reduced BW and fat deposition in offspring piglets. On the contrary, low-CP diets with in-feed AA supplementation (Lys, Met, Thr, and Trp) during gestation in primiparous gilts led to reduced ADG from days 91 to 110 of pregnancy, as well as urinary N excretion at different gestational stages, albeit not affecting the feces microbiome [82]. Shortly after, the same authors, in a similar experiment with sows, found that a CP reduction from 13 to 10% with in-feed AA supplementation (Lys, Met, Thr, and Trp) led to a reduced N excretion in feces and urine, a lower N retention, and an unchanged N retention ratio [83]. Using a different approach, Wu et al. [84] studied a combination of sequences of high and low CP dietary contents at different times of the day on sow performances and milk and plasma lipid profiles. The authors found that the maternal two-meal feeding sequences with varying CPs improved milk production and the milk lipid profiles of sows, ultimately contributing to piglet health and performance. These results are, however, difficult to interpret in the framework of this review, as the two experimental groups received a combination of high- and low-CP-content diets and there was no group receiving an exclusively low-CP-content diet. When AA supplementation is conducted, most of the previously stated negative effects of low CP dietary levels seem to be decreased. For instance, Figueroa et al. [85] evaluated the effects of different CP (between 15, 14, 13, 12, and 11%) contents in AA-supplemented diets (Lys, Met, Thr, and Trp) on growing breeding gilts, and reported that dietary CP affected growth performance, feed efficiency, fat-free lean gain, *longissimus* muscle area, plasma urea, and plasma concentrations of most essential AAs. They also suggested that other AAs (e.g., Ile and Val) might limit growth performance when the CP contents are reduced in 4%. On the contrary, low CP (from 17.5 to 13.5%) dietary contents supplemented with AAs (Lys, Met, Thr, and Val) did not affect litter ADGs in addition to AA transporters and/or milk protein gene expression in lactating sows, albeit they did increase mammary transport efficiency [86]. In another experiment, Huber et al. [87] evaluated the effect of decreasing CPs in the diet (16 to 14%), adding synthetic AAs (Lys, Ile, Met, Thr, Val, and Trp) in lactating sows. The authors showed that the low CP level improved N retention and N utilization efficiency for milk protein production in peak lactation (being less pronounced in early lactation), decreased loin area, and tended to increase litter growth rate, while milk composition was unaffected, albeit milk production increased in peak lactation.

## 5. Physiological Implications of Dietary CP Reduction

There are several studies addressing this topic. Many address aspects such as gene expression in tissues of economic relevance, namely the muscle, or of physiological importance, namely the intestines or the liver. The majority point to a relevant downregulation of metabolic pathways such as protein synthesis, lipid metabolism, or AA and peptide transporters. The recent advent of omics-based technologies in animal science research [88] has allowed for a broader and in-depth analysis of the different biochemical pathways that are affected by dietary CP reduction, in clear contrast with earlier studies that were very focused on a specific set of genes or regulatory pathway. The existence of contrasting results between experiments is particularly noticeable in piglets, which could be considerable expectable given the increasing growth rates and the different post-weaning stresses that challenge these types of animals.

In piglets, this subject has been addressed at different levels, focusing on different tissues. Earlier studies from the late 2000s focused on protein synthesis. Deng et al. [89] worked with diets containing 20.7, 16.7, or 12.7% CP, first reporting that a decrease in CP levels led to a decrease in protein synthesis in different organs (pancreas, liver, kidney, and skeletal muscle), ultimately inhibiting the mTOR signaling pathway that leads to cell differentiation and growth. These results are in accordance with those of Yin et al. [90], who looked at several organs (skeletal muscle, liver, the heart, kidney, pancreas, spleen, and stomach), and who determined that both a decrease in protein synthesis and mTOR signaling pathway inhibition could be minimized using increasing levels of Leu supplementation in the small intestine, kidney, and pancreas. Another topic that has been extensively studied in piglets is the intestinal expression of AA and peptide transporters. Zhang et al. [91] compared 20.9 vs. 17.1% CP and BCAA supplementation (Ile, Leu, Val) diets in Duroc × (Large White × Landrace) piglets and studied AA and peptide transporter gene expression in the intestine, observing that CP restriction led to restored small intestine villous heights if BCAAs were added. This same trend was recorded in the AA and peptide transporters studied. These authors concluded that BCAAs (especially Leu) may be necessary to maintain normal intestinal development and the physiological absorption of AAs via regulating the expression of intestinal AAs and peptide transporters in the small intestine. Other researchers analyzed the expressions of several AA transporters in the jejunum of Duroc × (Landrace × Yorkshire) female piglets fed with 14, 17, and 20% CP levels with the supplementation of several in-feed AAs. The authors reported that AA transporter gene expression, in the *Longissimus dorsi* muscle, was decreased because of the CP reduction and that AA supplementation could revert it, albeit not at the lowest CP content studied (14%) [92]. Li et al. [93] showed the effect of low (14%), medium (17%), and high (20%) CP levels, confirming an effect on intestinal AA transporter gene expression, intestinal morphology and mucosal barrier function. The authors also found that dietary CP levels could not be reduced by more than 3% in consideration of the maladaptive changes to small intestinal morphology and pepsin activity in weaned piglets. In contrast, other researchers [94] were not able to detect morphology changes in the jejunum of piglets fed with decreasing CP contents, albeit there were changes in gene expression, particularly transporters, and overall changes in the microbiome. This is consistent with the results by Tian et al. [95], where decreased dietary CP led to the reduced transcript abundance of nutrient transporters. On the contrary, Yu et al. [96], working with CP reductions from 20 to 14%, observed decreased villus heights and lower ratios of villus heights to crypt depths in piglets, although there were no changes in gastrointestinal hormones or jejunum microbiota. Finally, Lynegaard et al. [97], conducting research using protein restriction and AA supplementation in Duroc × (Danish Landrace × Danish Yorkshire) piglets and using transcriptomic analyses, observed major changes in the jejunum of the animals. These included the downregulation of several genes related to the immune system, protein metabolism, cell cycle mitosis, signal transduction, gene expression, or intracellular signaling, consistent with the growth reduction observed.

The colon microbiome also varies with decreases in dietary CP contents. Indeed, Yu et al. [96] did not observe any differences in the microbiome’s composition, whereas other researchers found reduced CP fermentation products and beneficial alterations in the proportions of *Firmicutes*, *Clostridium*, *E. coli*, and *Lactobacillus* clusters, in addition to a downregulation in the expression of pro-inflammatory cytokine genes [98]. Similar conclusions were also drawn by Limbach et al. [22], who overall concluded that CP reduction was an interesting way to reduce inflammation and fecal scores in the post-weaning piglet, and also by Tian et al. [99], who observed marked changes in the cecum microbiome of piglets fed with decreasing CP levels, with a consequent improvement in caecal barrier function.

Muscle tissue is also affected by dietary CP reduction in piglets. Wang et al. [100] tested 14, 17, and 20% dietary CP and found that several AA transporter gene expressions in the *Longissimus dorsi* muscle, albeit not the mTOR signaling pathway, were increased in the lowest CP level group, who showed a decrease in protein synthesis and muscle growth. These results contrast with those of Yin and colleagues [101], who concluded that dietary CP restriction downregulated AA transporters and the mTOR signaling pathway in the muscles of piglets. In fact, in piglets, the intestine is perhaps the tissue most affected by dietary CP reduction. Yan et al. [102] conducted an in-depth proteomics study on the *Longissimus dorsi* muscle of piglets fed adequate (20%) or low CP levels (17%, supplemented with Lys, Met, Thr, and Trp) using an iTRAQ-based approach. In that experiment, over 1300 proteins were quantified, showing differential accumulation in approximately 10% of the muscle proteome. As expected, the CP level significantly altered metabolic pathways that included lipids, carbohydrates, and AA metabolisms, showing higher accumulation in the control animals. Interestingly, the oxidative phosphorylation pathway was consistently downregulated in the muscles of low-CP-diet-fed animals, which the authors related to the limitations of some specific AA other than the supplemented Lys, Met, Thr, and Trp. This would, in turn, reduce the requirements of ATP produced by the mitochondria, and one could speculate that it would likely be highlighted in lower ADGs in piglets fed low-CP diets, regardless of AA supplementation, and when compared to controls, as described in Section 2. Nevertheless, as the authors do not provide data on the BWs of piglets, such information cannot be inferred. Low dietary CP levels also affect the piglet muscle metabolome. Conducting research using 18, 16, 14, and 12% CP levels in Shaziling (a Chinese fatty breed) pigs, Zheng et al. [103] studied the metabolites of the *Longissimus thoracis* muscle, in addition to several meat quality traits. The authors determined that some pork quality traits were significantly altered in animals fed low-CP diets, especially those related to color attributes, as well as several metabolites. The metabolomics results showed, furthermore, a clear separation in the two-dimensional scatterplot between lower and higher CP levels and, finally, diminished concentrations of the metabolites Danazol, N,N-dimethyl-Safingol, and cer(d18:0/14:0) were related to improved meat quality traits, ultimately suggesting that long-term CP restriction leads to improvements in pork quality. It is noteworthy to mention that, in other tissues of higher physiological relevance, such as the liver, the effect of low CP levels in the lipid metabolism is quite evident. Indeed, it has been observed that a low level of CP in the diet (14%) leads to significant changes in lipogenesis, lipolysis, oxidation, and gluconeogenesis pathways when compared to higher levels (17 or 20%) [104]. The blood metabolome is also significantly affected by dietary CP content (18 vs. 12%), as demonstrated by Spring et al. [21], who showed that N, starch and sucrose, and AA (Leu, Ile, and Val) metabolisms and biosynthesis in over 35 blood metabolites were significantly influenced, concurring with previous studies. Interestingly, maternal protein restriction also leads to changes in the blood metabolome of piglets, particularly serum iron levels in male weaning piglets [105].

In growing pigs, the physiological effects of low dietary CP levels have also been extensively studied. In an earlier study carried out by Ramsay and Mitchell [106], the authors studied the effect of 18 vs. 12% CP contents on uncoupling protein expression in several porcine tissues (muscle, liver, and adipose tissues). They suggest that low CP levels have interesting consequences by reducing lipoperoxidation and reactive oxygen species in these mitochondrial transporters, in addition to fatty acid transport. The muscle is also a widely studied tissue in growing pigs fed with low CP contents. Therefore, Wang et al. [107] showed that low-CP diets led to an upregulation of intra-muscular lipogenic genes and a downregulation of lipolytic genes in Wujin pigs, a Chinese fatty breed, consistent with higher IMF deposition in these animals. These results are consistent with others [108] who observed an increase in IMF and an upregulation of lipogenic genes as a consequence of low-CP diets in European Duroc × (Landrace × Yorkshire) genotypes. Low dietary CP levels have additionally been associated with the upregulation of mRNA expression of AA transceptors (dual-function AA transporters/receptors) in order to enhance the absorption of free AAs [107], as well as the AA metabolism, the mTOR signaling pathway, and protein synthesis in the skeletal muscles of growing pigs [101].

The effect of low dietary CP levels has also been studied in the intestines of growing pigs. He et al. [109] studied ileum gene expression in growing pigs fed ideal and below-ideal CP levels and found no differences between the groups. Similarly, low CP levels have also been found not to lead to changes in jejunum digestive enzyme activity [110]. On the contrary, both ileal and colon microbiomes are severely affected by medium–lower dietary CP levels that ultimately strengthen beneficial microbial populations and suppress harmful bacterial growth, as detailed by Chen et al. [111]. It has been reported that decreased CP levels increase microflora diversity and ultimately enhance animal health and CP digestibility in jejunum and cecum [100], in ileum, cecum, and the colon [112], in the colon [113], and in ileum and fecal microbiomes [114]. Finally, in growing pigs, low CP contents have also been associated with higher calcium and phosphorous digestibility [115], although lower levels of these minerals were retained when growing Iberian pigs (from 15 to 50 kg BW) were fed low-CP diets (10.1 vs. 19.2%) [64].

Contrary to piglets or growing pigs, physiological studies about the effects of low dietary CP levels on finishing pigs are very scarce. Indeed, in an earlier study, Weber et al. [116] found no effect on serum adiponectin and leptin concentrations, nor on the expression of genes such as acyl-CoA oxidase or fatty acid synthase in the hepatic tissue when pigs received 10.9 or 13.1% CP in the late phase and were slaughtered at 130 kg BW. More recently, Madeira et al. [117] compared 13.1 vs. 17.1% CP in both lean and fatty genotypes of finishing pigs (Large White × (Landrace × Pietrain) vs. Alentejano purebred), concluding that, albeit there were significant changes in the hepatic metabolism as a consequence of the genotype, no differences were found for lipogenesis as a result of dietary CP reduction. On muscle tissue, it has also been detected that a CP reduction from 14.2 to 11.1% led to increased expression of μ-calpain in skeletal muscles, but had no influence on gene expression of calpastatin, in addition to increased IMF content and pork tenderness [118]. More recently, low-CP diets (13.0 vs. 16.0%) have been associated with major changes in the muscle proteome of finishing pigs slaughtered at 94 kg BW [119], particularly proteins involved in muscle contraction and structural constituents of the cytoskeleton (upregulated in animals fed low-CP diets) and enzymes involved in energy metabolism (downregulated in animals fed low-CP diets). Similarly, regarding both piglets and growing animals, in finishing pigs, feeding low-CP diets (12 vs. 17%) has also been found to affect the microbiome and metabolome of different compartments of the gastro–intestinal tract, ultimately improving gut barrier function via the colonization of beneficial bacteria [120].

## 6. The Reduction in CP Contents in Swine Diets: An Overview of the Environmental Effects

Reductions in dietary CP contents and adequate AA supplementation are often suggested as means to lower emissions from the pig production sector. This topic has been extensively addressed through different studies, either focused on the different stages of pig production or on specific pollutants or environmental aspects. Although opposing results may be found in the literature, most studies find a significant reduction in pollutant emissions as a consequence of lowering the CP contents of pig feeds, particularly NH_3_. The subject has recently been reviewed [121,122]. As such, only the major effects are highlighted in this section.

**Figure 4:**
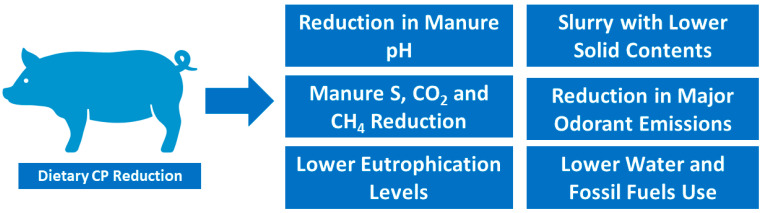
Major benefits associated with a dietary CP reduction strategy at the environmental level.

Interestingly, in an earlier study [123], the authors found that a dietary CP reduction (between 16.5, 14.5, and 12.5%) with a constant Lys, Met+Cys, Thr, and Trp content decreased NH_3_ emission by 10–12.5% for each percent decrease in dietary CP. In another study, Clark et al. [124] contrasted high- and low-CP diets (16.8 vs. 13.9%) regarding their effect on odor and GHG emissions. The authors found that manure from high-CP diets had higher sulfur concentration and pH, whereas CO_2_ and CH_4_ emissions from pig manure increased with lower dietary CP contents, which may be attributed to the lower growth performance of the pigs. On the contrary, Panetta et al. [125] found that fecal N and ammonia emission rates from pig manure decreased with CP contents. Other authors [126,127] obtained similar results for NH_3_ emissions and odor emission rates. In a companion paper, the latter team [128] found that a CP content reduction from 15 to 12% led to no changes in odor emission, odor intensity, or GHG concentration from the manure, albeit NH_3_ emission was reduced. Similar results were also obtained in finishing pigs [129]. Consistently, reducing the CP contents in feeds through the whole pig production chain led to reduced NH_3_ emissions and CH_4_ concentrations in manure [130], albeit increasing the hydrogen sulfide emissions [131]. Pigs fed with low CP contents (between 14 and 16% vs. 18%) have also been found to produce slurry with lower total solid and volatile solid contents, in addition to lower CH_4_ emissions [132]. These results contrast with those of Seradj et al. [133], who found no changes in the microbiome nor in the CH_4_ and NH_3_ emissions of pigs as a consequence of a 2% CP content reduction. Regarding this last study, it is interesting to point out that CH_4_ and NH_3_ emissions seem to be genotype dependent. Indeed, Duroc pure-bred animals had higher CH_4_ (two times) and NH_3_ (six times) emissions (in both cases, measured in g/animal/day) in comparison to leaner cross-bred animals, albeit the latter were heavier than the former [133]. More recently, Trabue et al. [134] detected a reduction in manure pH, total solids, total N, total sulfur, and concentrations of volatile fatty acids and phenol compounds, which are major odorants emitted from manure changes in gas emissions, with a reduction in feed CP content (between 17.6, 14.8, and 8.7%). The authors established between an 8.9 and 4.2% decrease for NH_3_ and odor emissions, respectively, per each CP unit decrease. Other benefits of CP reduction and consequent NH_3_ emissions include a lower exposure of workers to this contaminant [135], which is in accordance with the results obtained by Le Dinh et al. [136]. More recently, Beckmüller et al. [137] determined that barrows fed a diet combining both N and P reductions led to reductions of between 28 and 15%, respectively, without losses in growth, highlighting the importance of feeding strategies in reducing the environmental impacts of pig production. In Figure 4, we show a diagram of the major effects of a dietary CP reduction strategy at the environmental level, as detailed in this section.

An increase in indigestible CP concentration in the ileum, caused by heat damage, increased fecal N excretion and induced shifts in microbial communities. The N and fiber utilization in the hindgut may be inhibited in an indigestible N-rich environment [138], which implies that dietary CP digestibility should be cautiously considered in some protein source feedstuffs when decreasing dietary CP content. Recently, evidence that pigs fed low-CP diets (from 16.3 vs. 20.7% at 25–50 kg BW to 10.4 vs. 14.2% at 100–130 kg BW) had a high N utilization efficiency and showed the least impact on acidification, terrestrial eutrophication, and water use among these feeding programs [139]. The environmental load of the overall impact may be dependent on the crop–livestock connection in the area. For example, in a previous work conducted in North America, high-CP diets (maize–soybean based with low in-feed crystalline AA levels) optimized growth performance and carcass composition with reduced impacts on climate change, freshwater eutrophication, and fossil fuel use compared to feeding programs maximizing by-product use (with dry-distilled grain inclusion) or reduced CPs with high in-feed crystalline AAs. However, under European conditions, multi-objective formulations, which considered the feed costs and environmental impacts calculated using life cycle assessments, reduced, at feed level, the impacts on climate change, non-renewable energy, acidification, and P demand, but sometimes increased the impacts on land occupation and eutrophication. These ecofriendly formulations reduced the proportion of cereals and oilseed meals into feeds (feed ingredients with high impacts under some European conditions), while the proportion of alternative protein sources, like peas, fava beans, or high-protein agricultural coproducts, increased [140].

## 7. Conclusions and Future Perspectives

The reduction in dietary CP contents in pig feeding is an interesting strategy that presents several pros and cons and has different applications according to the production objectives and, above all, the growing/productive phase of the animal. Generally speaking, and despite the variety of results found in the literature, a simple CP reduction from 2 to 5% leads to production losses in all swine types. Indeed, and while breeders (boars and highly prolific sows) may display yielding limitations, the piglets and growing and finishing pigs may show lower ADGs and higher FCRs that significantly affect the economic returns from pig production. This makes the strategy particularly debatable and, from a productive point of view, highlights the need to supplement feeds with synthesized amino acids.

Indeed, production losses inherent to CP reduction can be mitigated when animals are supplemented with essential AAs (except in organic production, where this does not apply), with the particular relevance of Lys, Met, Thr, and/or Trp. Under these situations, CP content may be decreased to values 3–5% lower than those suggested by most animal nutrition systems, particularly in the pork meat production stages. This strategy has to be necessarily coupled to AA supplementation, with particular relevance to those AAs abovementioned and within the reference values suggested by the pig nutrition guidelines as recommended by the different nutrition systems. Indeed, the NRC [13] set the dietary N requirements as approximately twice the amount of the essential (or partly essential) digestible AAs required (in alphabetic order: Arg, His, Ile, Leu, Lys, Met, Met+Cys, Phe, Phe+Tyr, Thr, Trp, and Val). This means that half of the dietary N supplied must be directed to meet essential AA requirements, whereas the other half of the N is used as a pool for non-essential AA synthesis. However, in many feeding systems, this additional N pool has an excessive safety margin.

Nevertheless, and despite a similitude of productive performances, numerous changes of a more physiological nature affect animals fed with lower CP contents. These include, for instance, and in earlier stages of development, a decrease in lipogenesis, an inhibition of the mTOR signaling pathway and subsequently hindering adipose and muscle tissue growth and cellular differentiation, as well as a decrease in the expression of AA transporter genes.

**Figure 5:**
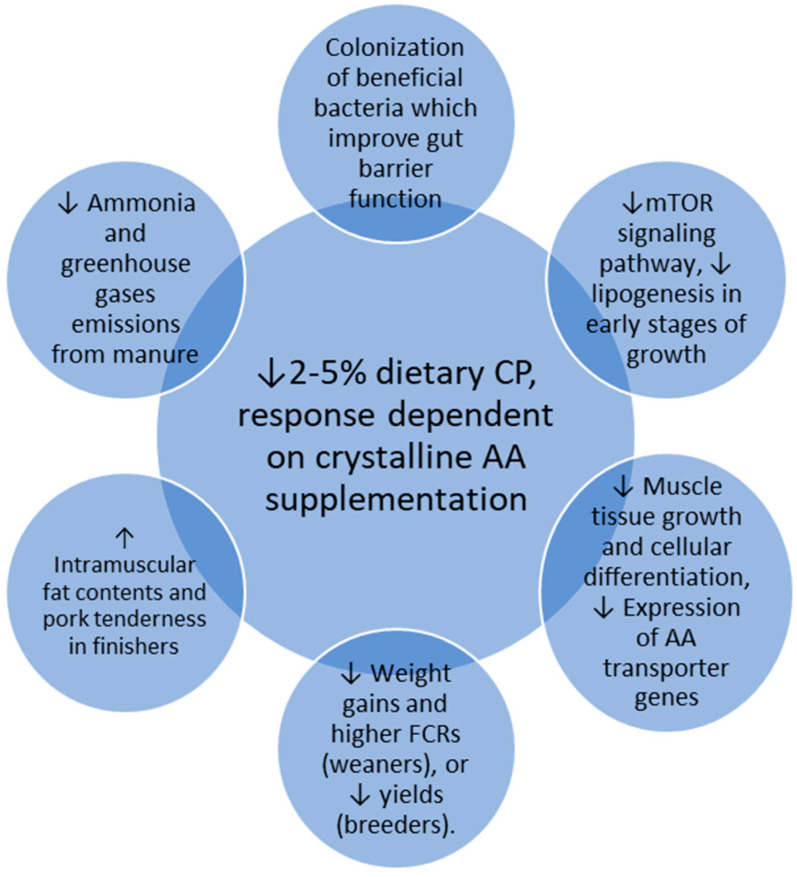
Major effects at the productive and physiological levels of a reduction of 2–5% in dietary CP, with a response dependent on crystalline amino acid supplementation.

In finishing animals, the situation is quite different, and lowering dietary CP contents may lead to an increase in IMF contents and pork tenderness. Indeed, it could be stated that piglets and breeders are perhaps the type of animals where the CP reduction strategy, regardless of AA supplementation, would be the most critical, whereas in growing and particularly in finishing pigs, it could prove to be a more interesting strategy from a productive point of view. In addition, dietary CP reduction, regardless of AA supplementation, seems to lead to interesting changes in the microbiomes of various gastrointestinal tract compartments in all types of animals. Such changes ultimately improve the gut barrier function via the colonization of beneficial bacteria, and are a major asset in the framework of the current challenges of pig production, particularly the limitations on the use of ZnO or antibiotics during the post-weaning phase. Another interesting and almost unanimous consequence of dietary CP reduction, also regardless of AA supplementation, is the environmental component, where 2–5% reductions lead to relevant reductions in NH_3_ and GHG emissions from manure, with clear benefits for the environment and workers’ conditions, as well as for animal welfare. The most-significant effects of a dietary CP reduction in pigs are summarized in Figure 5.

As stated, reducing dietary CP contents seems to be more promising during the finishing phase. Indeed, this is the phase where AA and protein requirements are lower and where animals have higher feed intakes. As such, it would be the phase where NH_3_ emissions reductions would be more relevant and where animal growth would be less affected. The finishing phase is, however, the least studied, particularly for pigs of less conventional genotypes (e.g., Duroc crosses) that are produced until higher market weights than those conventionally used (e.g., 120–130 kg). This may be a consequence of the difficulty in working with large animals compared to, for instance, piglets. It is thus of the utmost importance to increase research outputs on the usefulness of the dietary CP reduction strategy in finishing pigs. The studied aspects should include, in addition to standard growth performance and pork traits, a special emphasis on the molecular aspects underlying such performances. This can be achieved, for instance, through omics-based studies targeting muscle, hepatic, or the adipose tissues.

Another interesting aspect clearly neglected in the research conducted so far are response differences between entire male and female pigs that are the most common pork types under castration practice limitations in many areas of the world. Indeed, both sexes are generally fed the same feed. However, they have different nutritional requirements and, as such, the dietary CP reduction strategy is likely to have different consequences for males and females. Additionally, and as the protein efficiency is a highly heritable trait (as recently determined [141]), it would also be interesting to focus research on the effects of reduced dietary CP in different genetic lines, particularly high-growth lean genotypes and heavier fattier Southern European breeds. Regarding Southern European breeds, it would be interesting to highlight the effects of the CP reduction strategy on the different pig production systems, contrasting, for instance, the two sexes, castration protocols, and different feeding strategies with particular relevance to traditional finishing diets and with a clear link to product (e.g., traditional hams and sausages) quality and acceptance by consumers. It would also be interesting to conduct an economic sensitivity analysis under different scenarios (feedstuff and pork prices) for all research on dietary CP reduction in order to ascertain to what extent the strategy profitable for the pig industry is in different agricultural economic contexts, contrasting, for instance, the Northern European, Southern European, North American, Chinese, or Southeast Asian realities. Finally, and with growing importance in the current environmental contexts and limitations, it is of the utmost relevance to couple studies that encompass aspects such as effluent management, greenhouse gas emissions, animal welfare, and animal physiology, in addition to classical nutritional and economic studies on the applicability of the strategy.

## Data Availability

Not applicable.
